# Erectile Dysfunction in Patients with Sleep Apnea – A Nationwide Population-Based Study

**DOI:** 10.1371/journal.pone.0132510

**Published:** 2015-07-15

**Authors:** Chia-Min Chen, Ming-Ju Tsai, Po-Ju Wei, Yu-Chung Su, Chih-Jen Yang, Meng-Ni Wu, Chung-Yao Hsu, Shang-Jyh Hwang, Inn-Wen Chong, Ming-Shyan Huang

**Affiliations:** 1 Division of Pulmonary and Critical Care Medicine, Department of Internal Medicine, Kaohsiung Medical University Hospital, Kaohsiung Medical University, Kaohsiung, Taiwan; 2 Graduate Institute of Medicine, College of Medicine, Kaohsiung Medical University, Kaohsiung, Taiwan; 3 Sleep Disorders Center, Kaohsiung Medical University Hospital, Kaohsiung Medical University, Kaohsiung, Taiwan; 4 Department of Internal Medicine, Kaohsiung Municipal Ta-Tung Hospital, Kaohsiung Medical University, Kaohsiung, Taiwan; 5 School of Medicine, College of Medicine, Kaohsiung Medical University, Kaohsiung, Taiwan; 6 Department of Neurology, Kaohsiung Medical University Hospital, Kaohsiung Medical University, Kaohsiung, Taiwan; 7 Division of Nephrology, Department of Internal Medicine, Kaohsiung Medical University Hospital, Kaohsiung Medical University, Kaohsiung, Taiwan; 8 Department of Renal Care, College of Medicine, Kaohsiung Medical University, Kaohsiung, Taiwan; 9 Department of Respiratory Care, College of Medicine, Kaohsiung Medical University, Kaohsiung, Taiwan; 10 Division of Geriatrics and Gerontology, Department of Internal Medicine, Kaohsiung Medical University Hospital, Kaohsiung Medical University, Kaohsiung, Taiwan; Osaka University Graduate School of Medicine, JAPAN

## Abstract

Increased incidence of erectile dysfunction (ED) has been reported among patients with sleep apnea (SA). However, this association has not been confirmed in a large-scale study. We therefore performed a population-based cohort study using Taiwan National Health Insurance (NHI) database to investigate the association of SA and ED. From the database of one million representative subjects randomly sampled from individuals enrolled in the NHI system in 2010, we identified adult patients having SA and excluded those having a diagnosis of ED prior to SA. From these suspected SA patients, those having SA diagnosis after polysomnography were defined as probable SA patients. The dates of their first SA diagnosis were defined as their index dates. Each SA patient was matched to 30 randomly-selected, age-matched control subjects without any SA diagnosis. The control subjects were assigned index dates as their corresponding SA patients, and were ensured having no ED diagnosis prior to their index dates. Totally, 4,835 male patients with suspected SA (including 1,946 probable SA patients) were matched to 145,050 control subjects (including 58,380 subjects matched to probable SA patients). The incidence rate of ED was significantly higher in probable SA patients as compared with the corresponding control subjects (5.7 vs. 2.3 per 1000 patient-year; adjusted incidence rate ratio = 2.0 [95% CI: 1.8-2.2], *p*<0.0001). The cumulative incidence was also significantly higher in the probable SA patients (*p*<0.0001). In multivariable Cox regression analysis, probable SA remained a significant risk factor for the development of ED after adjusting for age, residency, income level and comorbidities (hazard ratio = 2.0 [95%CI: 1.5-2.7], *p*<0.0001). In line with previous studies, this population-based large-scale study confirmed an increased ED incidence in SA patients in Chinese population. Physicians need to pay attention to the possible underlying SA while treating ED patients.

## Introduction

Sleep apnea (SA) is the most common form of sleep disordered breathing characterized by repetitive cessation of breathing during sleep, usually associated with intermittent hypoxia and sleep fragmentation [1–3]. Results from Wisconsin Sleep Cohort study estimate the prevalence of SA was 9% for women and 24% for men [4]. The diagnosis of SA is usually made by frequent apnea/hypopnea events during sleep, i.e. increased apnea-hypopnea index (AHI), on nocturnal polysomnography (PSG) [5]. More than 90% of patients have obstructive sleep apnea (OSA), characterized by recurrent upper airway collapse, while less than 10% of patients have central sleep apnea (CSA), which is related to losing neurological drives of respiratory effort [1,2].

Erectile dysfunction (ED), defined as the inability to obtain and maintain an erection sufficient for satisfactory sexual intercourse, is both highly prevalent and inadequately treated [6]. Over 152 million men were estimated to have ED in 1995, and this prevalence was projected to an estimation of 322 million by 2025 [7]. Because sexual health is important for overall wellbeing, ED is associated with significantly lower quality of life [8]. More importantly, ED has been recognized as an important sentinel event for peripheral artery disease, coronary artery disease (CAD), stroke and even all cause-mortality [9–11].

SA is associated with various diseases such as hypertension, ischemic heart disease, diabetes mellitus, stroke, and hormonal dysfunction [12,13]. Many previous studies have demonstrated that patients with SA have significant risk for erectile dysfunction (ED), and some studies have also shown high prevalence of SA in men with ED [6,14–20]. Although the association between SA and ED has been widely discussed, however, this association has not been confirmed in a large-scale cohort study. We therefore conducted this large population-based cohort study to further strengthen the association between SA and ED.

## Methods

### Data Sources

The Taiwan National Health Insurance (NHI) has covered ambulatory care, hospital inpatient care, dental services, and prescription drugs since March, 1995. The NHI coverage rate was 96.16% of the whole population of 23 million in 2000 and rose to 99% by the end of 2004 [21]. The NHI medical reimbursement claims database is managed by the National Health Research Institutes in Taiwan. The dataset used for this study is Longitudinal Health Insurance Database 2010 (LHID2010), a cohort of 1 million randomly sampled subjects in the NHI system in 2010 and included their reimbursement information until the end of 2010. Patient identification numbers were encrypted for confidentiality. The study protocol was approved by the Kaohsiung Medical University Hospital Institutional Review Board (KMUH-IRB-EXEMPT-20130034).

### Sleep Apnea Cohorts

**Fig 1:**
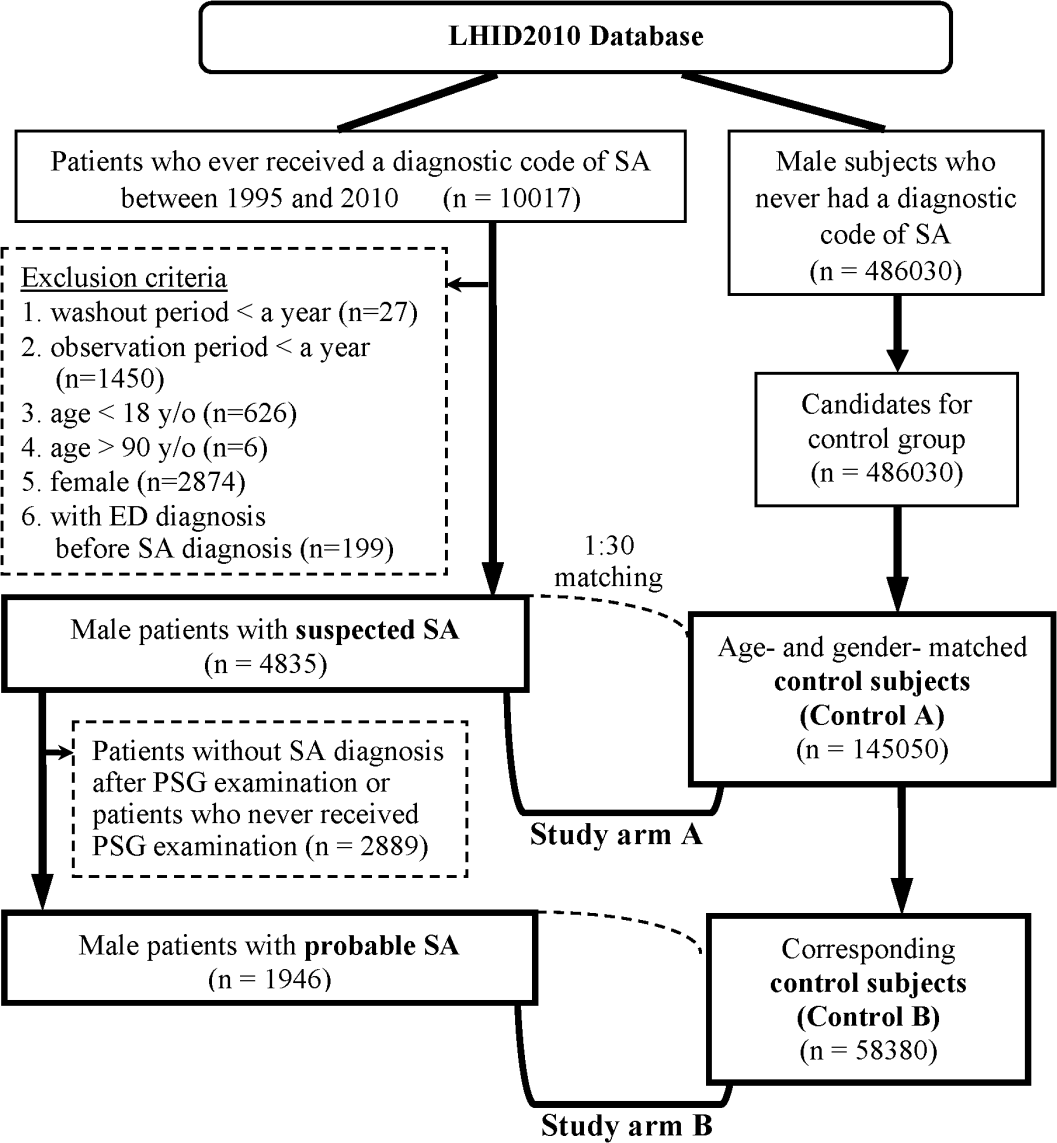
Algorithm for identifying the study population. (Abbreviations: SA = sleep apnea; ED = erectile dysfunction; PSG = polysomnography).

A total of 4,835 male patients were identified as the “suspected SA cohort” by the algorithm (Fig 1). In brief, patients with SA diagnosis between March 1, 1995 and December 31, 2010 were identified initially. The International Classification of Diseases, Ninth Revision, Clinical Modification (ICD-9-CM) codes of 327.20, 327.21, 327.23, 327.27, 327.29, 780.51, 780.53, and 780.57 (Table A in S1 File) were used for diagnosis of SA [1–3]. The ICD-9-CM codes of 607.84 and 302.72 were used for diagnosis of ED [22,23]. Each patient was followed from the date of his initial SA diagnosis, defined as the index date, to either their first diagnosis of ED, end of the study period, or termination of the record because of death or withdrawal from the insurance program, whichever came first. In order to increase the likelihood of including only newly diagnosed SA cases, patients with washout periods (from NHI enrollment to the index date) less than a year were excluded. Patients with diagnosis of ED before the index date were excluded. Patients with follow-up periods less than a year were excluded to ensure enough follow-up periods. Besides, patients younger than 18 years old or elder than 90 years old on the index date were also excluded.

From the 4,835 patients with suspected SA, those who had ever received PSG examination and remained having SA diagnosis after PSG were further extracted; these 1,946 patients were defined as the “probable SA cohort.”

### Control Cohorts

For each patient with suspected SA, thirty age-matched control subjects were randomly selected. The control subjects were given the same index date as their corresponding SA patients. During the matching process, the same exclusion criteria for the SA patients were also applied while selecting control subjects to ensure enough washout periods and follow-up periods and the absence of any ED diagnosis before the index date. Finally, 145,050 control subjects (control A cohort) were matched to the patients with suspected SA (suspected SA cohort), and those who matched to the patients with probable SA (probable SA cohort) were also extracted as control B cohort (n = 58,380). As the SA patients, the control subjects were followed from the index date to either the development of ED, end of the study period, or termination of the record because of death or withdrawal from the insurance program, whichever came first.

### Criteria and definitions of variables

The endpoint of this study was the development of ED, defined by the first appearance of ED diagnosis. To increase the reliability of the diagnosis, only those with ED diagnosis for at least three times in the ambulatory claim database or at least once in the inpatient claim database were considered having ED.

The presence of comorbidity is identified by the presence of any corresponding diagnostic codes before the index date in the claim databases and confirmed by the presence of the codes for at least three times in the ambulatory claim database or at least once in the inpatient claim database. Based on the comorbidities, Charlson Comorbidity Index (CCI) score was calculated [24].

### Statistical analysis

This study composed of two study arms with identical statistical analyses. Study arm A compared the data between the suspected SA cohort and control A cohort; study arm B compared the data between the probable SA cohort and control B cohort.

First, the demographic data, comorbidities and CCI score were compared between the SA cohorts and the corresponding control cohorts using the Pearson χ^2^ test for categorical variables or Student’s t test for continuous variables, as appropriate. The ED incidence rate was calculated as the number of ED developed during the follow-up period divided by the total person-year. The ED incidence rates of SA cohorts and control cohorts were further compared by calculating the incidence rate ratio (IRR), defined as the ratio of the ED incidence rates of SA cohort and the corresponding control cohort. The 95% confidence intervals (95% CIs) for the IRRs were estimated under the assumption that the observed number of ED followed a Poisson probability distribution. Stratified analyses were also performed, by classifying the subjects with age group, residency, income level or the presence of any comorbidity. The adjusted IRRs were calculated by multivariable analyses adjusting for age, residency, income and the presence of various comorbidities (except for the variable used for stratification). Cumulative incidence of ED was calculated and compared with Kaplan-Meier method and log-rank test. To further assess the effect of SA, multivariable Cox proportional hazards regression analyses were performed with adjustment of age, residency, income level and comorbidities. After excluding the subjects having events in the initial 1–5 years of the follow-up period, sensitivity analyses were performed on the remaining SA patients and ten randomly-selected corresponding control subjects with the new follow-up periods starting from 1–5 years after the index dates (Table D in S1 File).

Extraction and computation of data, data linkage, processing and sampling and all statistical analyses were performed using SAS system (version 9.3 for Windows, SAS Institute Inc., Cary, NC, USA). The statistical significance level was set at a two-sided *p* value of < 0.05.

## Results

Totally, 4,835 male patients with suspected SA, including 1,946 patients with probable SA, were identified and matched to age-matched control subjects by the algorithm (Fig 1). The baseline characteristics of study cohorts are presented in Table 1. The mean (± standard deviation (SD)) age was 45.6±14.2 years in the suspected SA cohort and the control A cohort; the mean (±SD) age was 46.4±13.1 years in the probable SA cohort and the control B cohort. As compared with the subjects in corresponding control cohorts, patients in the suspected SA cohort and probable SA cohort had more comorbidities, in terms of heart diseases, major neurological disorders, chronic pulmonary diseases, connective tissue diseases, peptic ulcer disease, liver disease, diabetes mellitus, renal disease and cancer (Table 1).

**Table 1:** Baseline characteristics of the study population.

	Study arm A			Study arm B		
	Control A	Suspected SA	*P* value	Control B	Probable SA	*P* value
N	145050 (100%)	4835 (100%)		58380 (100%)	1946 (100%)	
Age (year), mean ± SD	45.6 ± 14.2	45.6 ± 14.2		46.4 ± 13.1	46.4 ± 13.1	
Age (year), n (%)						
≤ 40	56940 (39%)	1898 (39%)		20400 (35%)	680 (35%)	
40 < age ≤ 50	38760 (27%)	1292 (27%)		16920 (29%)	564 (29%)	
> 50	49350 (34%)	1645 (34%)		21060 (36%)	702 (36%)	
Residency						
Northern Taiwan	48356 (33%)	1809 (37%)	<0.0001	19133 (33%)	800 (41%)	<0.0001
Other areas	96694 (67%)	3026 (63%)		39247 (67%)	1146 (59%)	
Monthly income (NT$), median(IQR)	21000 (1249–40100)	25200 (1249–45800)	<0.0001	21000 (1249–42000)	30300 (17280–50600)	<0.0001
Monthly income (NT$), n (%)						
≤ 24000	86823 (60%)	2401 (50%)	<0.0001	33925 (58%)	850 (44%)	<0.0001
> 24000	58227 (40%)	2434 (50%)		24455 (42%)	1096 (56%)	
Wash-out period (day), median (IQR)	11.2 (8.6–13.1)	11.3 (8.7–13.1)	0.0269	11.4 (8.9–13.1)	11.4 (9.1–13.1)	0.0561
Follow-up period (day), median (IQR)	4.3 (2.5–6.7)	4.3 (2.5–6.7)	0.6427	4.2 (2.5–6.4)	4.1 (2.5–6.4)	0.5845
CCI score, mean ± SD	0.7 ± 1.3	1.2 ± 1.7	<0.0001	0.7 ± 1.3	1.3 ± 1.6	<0.0001
CCI score, n (%)						
= 0	93086 (64%)	2113 (44%)	<0.0001	36910 (63%)	766 (39%)	<0.0001
= 1	28002 (19%)	1240 (26%)		11579 (20%)	535 (27%)	
≥ 2	23962 (17%)	1482 (31%)		9891 (17%)	645 (33%)	
Underlying diseases, n (%)						
Heart disease	2740 (2%)	200 (4%)	<0.0001	1051 (2%)	87 (4%)	<0.0001
Myocardial infarction	1075 (1%)	63 (1%)	<0.0001	431 (1%)	25 (1%)	0.0062
Congestive heart failure	1934 (1%)	156 (3%)	<0.0001	735 (1%)	68 (3%)	<0.0001
Peripheral vascular disease	1024 (1%)	42 (1%)	0.1854	425 (1%)	18 (1%)	0.3167
Major neurological disorder	6955 (5%)	432 (9%)	<0.0001	2769 (5%)	185 (10%)	<0.0001
Cerebral Vascular disease	6598 (5%)	409 (8%)	<0.0001	2608 (4%)	176 (9%)	<0.0001
Dementia	624 (0%)	48 (1%)	<0.0001	217 (0%)	16 (1%)	0.0016
Hemiplegia	913 (1%)	40 (1%)	0.0886	397 (1%)	16 (1%)	0.4543
Chronic pulmonary disease	18230 (13%)	1266 (26%)	<0.0001	7363 (13%)	570 (29%)	<0.0001
Connective tissue disease	1034 (1%)	54 (1%)	0.0011	437 (1%)	22 (1%)	0.0564
Peptic ulcer disease	22658 (16%)	1219 (25%)	<0.0001	9345 (16%)	502 (26%)	<0.0001
Liver disease	16850 (12%)	1081 (22%)	<0.0001	7076 (12%)	483 (25%)	<0.0001
Diabetes mellitus	10496 (7%)	487 (10%)	<0.0001	4390 (8%)	212 (11%)	<0.0001
Renal disease	3020 (2%)	184 (4%)	<0.0001	1239 (2%)	71 (4%)	<0.0001
Cancer	3344 (2%)	199 (4%)	<0.0001	1330 (2%)	77 (4%)	<0.0001
Others (non-CCI items)						
Hypertension	26545 (18%)	1629 (34%)	<0.0001	11011 (19%)	763 (39%)	<0.0001

Abbreviation: SA = sleep apnea; CCI = Charlson Comorbidity Index.

Patients with suspected SA had significantly higher ED incidence rate than subjects in control A cohort did (4.4 vs. 2.3 per 1000 patient-year; adjusted IRR = 1.7 [95% CI: 1.6–1.8], *p*<0.0001); significantly higher ED incidence rate was also noted in patients with probable SA as compared with subjects in control B cohort (5.7 vs. 2.3 per 1000 patient-year; adjusted IRR = 2.0 [95% CI: 1.8–2.2], *p*<0.0001) (Table 2). On stratified analyses, patients with SA had significantly higher ED incidence rate as compared with subjects in the corresponding control cohort in all strata (Table 2).

**Table 2:** Incidence rate of erectile dysfunction after the index date in each group.

	Study arm A										Study arm B									
	Control A				Suspected SA				Crude IRR [95% CI]	Adjusted IRR [95% CI]	Control B				Probable SA				Crude IRR [95% CI]	Adjusted IRR [95% CI]
	N	ED	PY	IR	N	ED	PY	IR			N	ED	PY	IR	N	ED	PY	IR		
**Whole study population**	145050	1614	705703.1	2.3	4835	103	23430.0	4.4	1.9 [1.8–2.1]^***^	1.7 [1.6–1.8]^***^	58380	643	277701.5	2.3	1946	52	9188.7	5.7	2.4 [2.2–2.7]^***^	2.0 [1.8–2.2]^***^
**Stratified analyses**																				
**Age**																				
**≤ 50**	95700	670	476028.0	1.4	3190	55	15787.5	3.5	2.5 [2.3–2.7]^***^	1.9 [1.8–2.1]^***^	37320	260	181850.4	1.4	1244	29	6003.0	4.8	3.4 [3.0–3.8]^***^	2.5 [2.2–2.8]^***^
**> 50**	49350	944	229675.1	4.1	1645	48	7642.4	6.3	1.5 [1.3–1.7]^***^	1.4 [1.2–1.6]^***^	21060	383	95851.1	4.0	702	23	3185.7	7.2	1.8 [1.5–2.2]^***^	1.6 [1.3–1.9]^***^
**Residents in**																				
**Northern Taiwan**	48356	675	251968	2.7	1809	49	9624.7	5.1	1.9 [1.7–2.1]^***^	1.7 [1.5–1.9]^***^	19133	263	97702.3	2.7	800	27	4304.1	6.3	2.3 [2.0–2.7]^***^	2.0 [1.7–2.4]^***^
**Other areas**	96694	939	453735.1	2.1	3026	54	13805.3	3.9	1.9 [1.7–2.1]^***^	1.6 [1.5–1.8]^***^	39247	380	179999.2	2.1	1146	25	4884.5	5.1	2.4 [2.1–2.8]^***^	2.0 [1.7–2.3]^***^
**Monthly income**																				
**≤ NT$24000**	86823	882	420118.2	2.1	2401	42	11524.7	3.6	1.7 [1.6–1.9]^***^	1.5 [1.4–1.7]^***^	33925	351	160024.6	2.2	850	18	3921.4	4.6	2.1 [1.8–2.5]^***^	1.7 [1.5–2.0]^***^
**> NT$24000**	58227	732	285584.9	2.6	2434	61	11905.2	5.1	2.0 [1.8–2.2]^***^	1.7 [1.6–1.9]^***^	24455	292	117676.9	2.5	1096	34	5267.2	6.5	2.6 [2.3–3.0]^***^	2.2 [1.9–2.5]^***^
**Comorbidity**																				
**No (CCI score = 0)**	93086	795	477442.7	1.7	2113	51	11079.9	4.6	2.8 [2.5–3.0]^***^	2.8 [2.6–3.1]^***^ ^v^	36910	281	185073.5	1.5	766	22	3955.5	5.6	3.7 [3.2–4.2]^***^	3.6 [3.1–4.1]^***^
**Yes (CCI score ≥1)**	51964	819	228260.4	3.6	2722	52	12350.0	4.2	1.2 [1.0–1.3]^**^	1.1 [1.0–1.3]^*^	21470	362	92628.0	3.9	1180	30	5233.2	5.7	1.5 [1.3–1.7]^***^	1.4 [1.2–1.7]^***^

The adjusted IRRs were calculated by multivariable analyses adjusting for age, residency, income and the presence of various comorbidities (except for the variable used for stratification).

*p<0.05

**p<0.01

***p<0.0001

Abbreviation: SA = sleep apnea; CCI = Charlson Comorbidity Index

N = number of patients; ED = number of patients with erectile dysfunction; PY = total patient-years

IR = incident rate, as expressed as ED incidence per 1000 patient-years; IRR = incidence rate ratio; CI = confidence interval.

**Fig 2:**
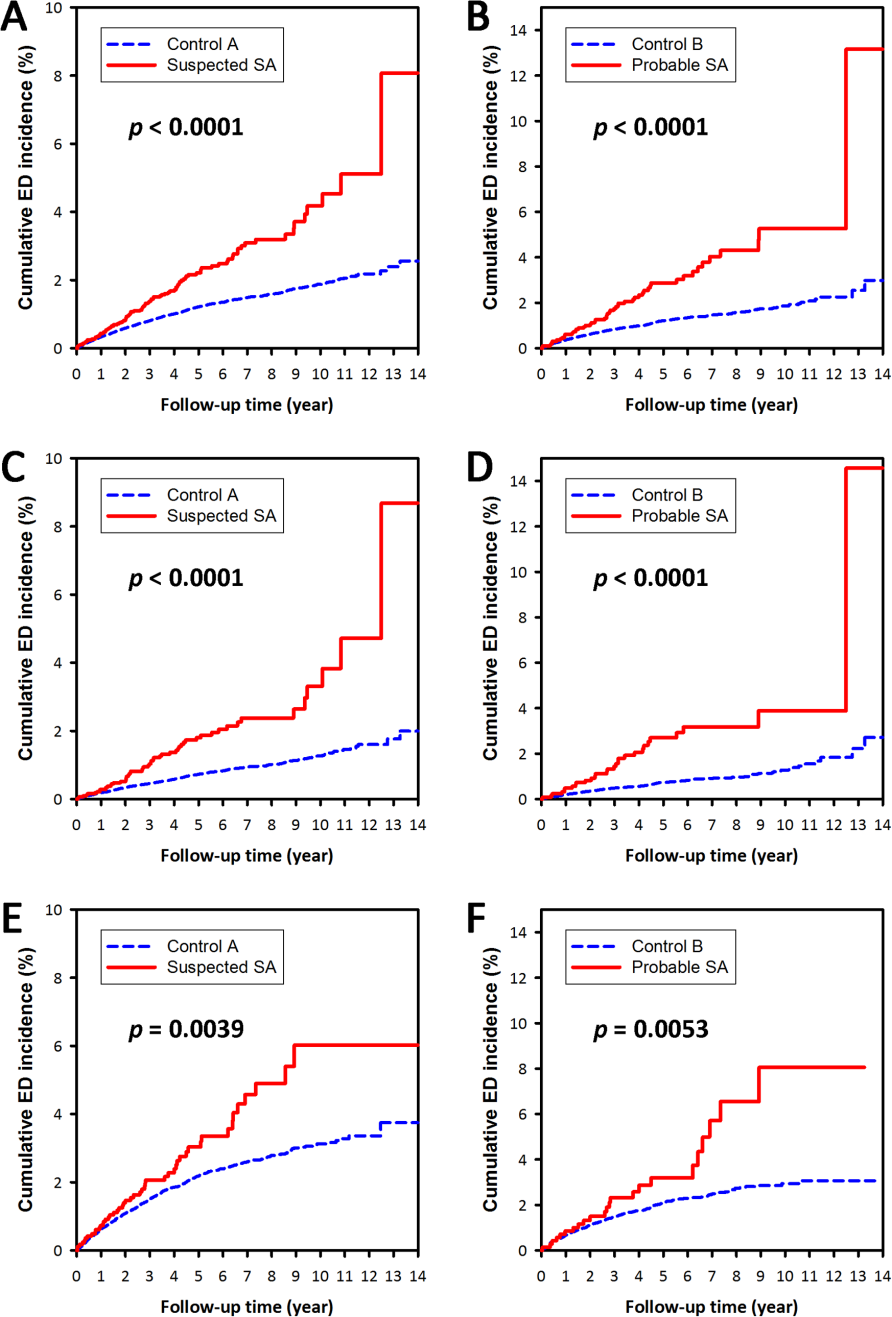
The cumulative incidences of erectile dysfunction (ED). The blue dashed lines and red continuous lines show the cumulative incidence of ED for the control cohort and the sleep apnea (SA) cohort, respectively. (A, C, E) study arm A (suspected SA vs. control A); (B, D, F) study arm B (probable SA vs. control B); (A, B) whole study population; (C, D) subjects ≤ 50 years old; (E, F) subjects >50 years old.

The cumulative ED incidence was significantly higher for both suspected SA cohort (as compared with control A cohort, *p*<0.0001) and probable SA cohort (as compared with control B cohort, *p*<0.0001) (Fig 2). On stratified analyses, the SA cohorts had a significantly higher cumulative ED incidence as compared with the corresponding control cohorts in strata of either subjects with age up to 50 years old, or subjects older than 50 years old (all *p*<0.01) (Fig 2).

**Fig 3:**
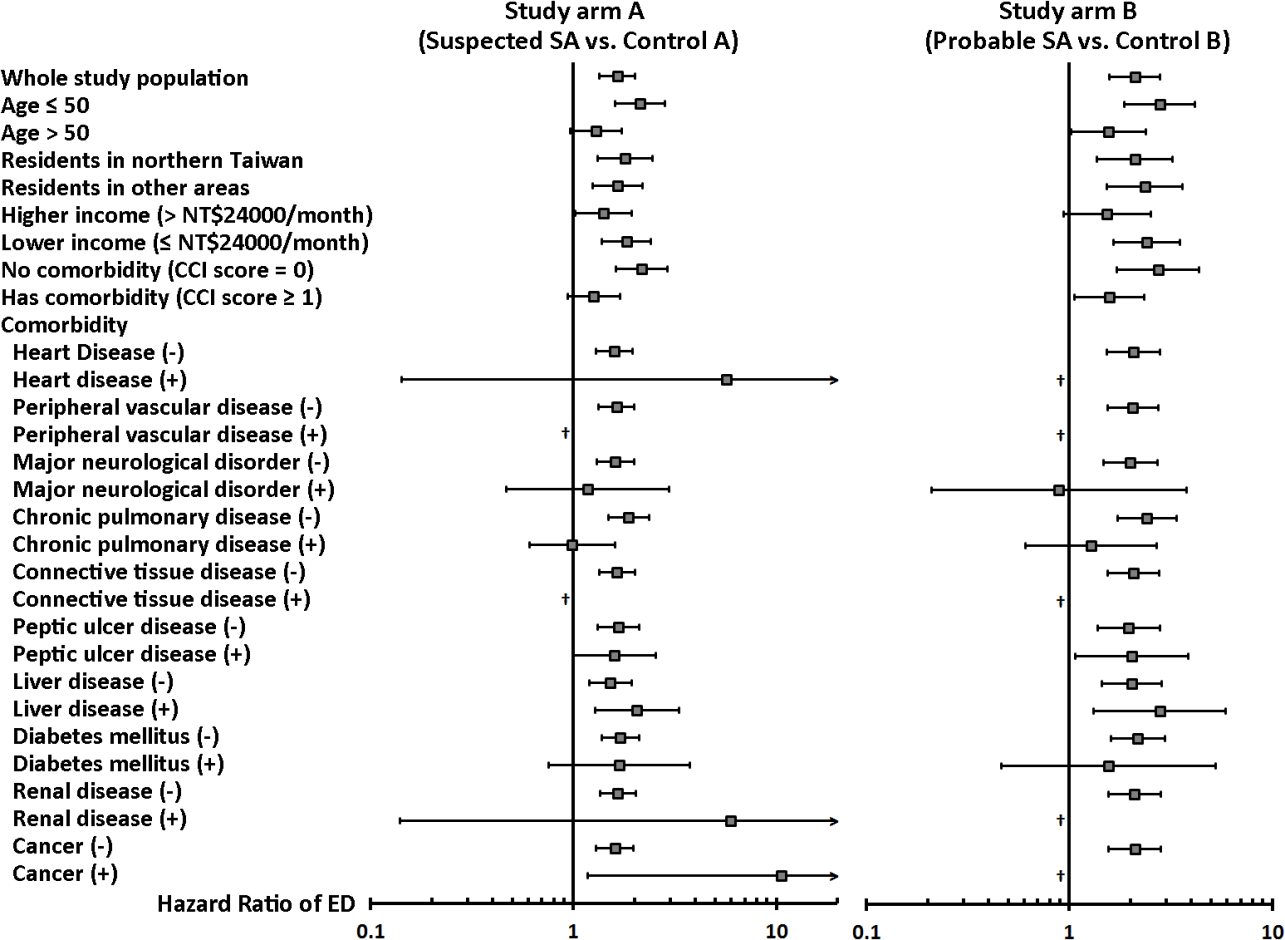
Stratified analyses of the multivariable Cox proportional hazards regression analyses. The results are presented with adjusted HRs (95% CI) of sleep apnea, which are adjusted for age, residency, income and the presence of various comorbidities (except for the variable used for stratification). *Abbreviations: SA = sleep apnea; CCI = Charlson Comorbidity Index; HR = hazard ratio; CI = confidence interval. †: Due to small sample size, hazard ratio cannot be estimated.

On multivariable Cox proportional hazards regression analyses comparing the ED incidences in probable SA cohort and control B cohort, SA was an independent risk factor for developing ED (hazard ratio [HR]: 2.0, 95% CI: 1.5–2.7, *p*<0.0001) (Table 3 and Table C in S1 File). The analysis comparing suspected SA cohort and control A cohort (study arm A) showed a consistent result (Table 3 and Table B in S1 File). On stratified analyses, the presence of either suspected SA or probable SA was associated with a higher risk for developing ED both in patients ≤ 50 years old and those > 50 years old (Fig 3). The increased ED risk related to SA was more significant in patients without comorbidities, whereas the effect of SA was modest in those with comorbidities.

**Table 3:** Multivariable Cox regression analysis of the factors contributing to erectile dysfunction – maximal models.

	Study arm A (Suspected SA vs. Control A)		Study arm B (Probable SA vs. Control B)	
	HR [95% CI]	*P* value	HR [95% CI]	*P* value
**SA patients vs. Control subjects**	1.7 [1.4–2.1]	<0.0001	2.0 [1.5–2.7]	<0.0001
**Age > 50 vs. Age ≤ 50**	2.7 [2.5–3.0]	<0.0001	2.3 [2.0–2.8]	<0.0001
**Residency (Northern Taiwan vs. Other areas)**	1.4 [1.3–1.6]	<0.0001	1.4 [1.2–1.6]	<0.0001
**Higher income (> NT$24000) vs. lower income (≤ NT$24000)**	1.4 [1.3–1.6]	<0.0001	1.3 [1.1–1.5]	0.0004
**Presence of underlying diseases:**				
**Heart disease**	0.7 [0.5–1.0]	0.0625	0.9 [0.6–1.4]	0.7094
**Peripheral vascular disease**	0.5 [0.3–1.1]	0.0700	0.4 [0.1–1.3]	0.1164
**Major neurological disorder**	1.1 [0.9–1.3]	0.5071	1.3 [1.0–1.7]	0.0779
**Chronic pulmonary disease**	1.1 [1.0–1.3]	0.0895	1.1 [0.9–1.3]	0.4791
**Connective tissue disease**	0.8 [0.4–1.3]	0.3522	0.7 [0.3–1.8]	0.4883
**Peptic ulcer disease**	1.0 [0.9–1.2]	0.8412	1.2 [1.0–1.4]	0.1063
**Liver disease**	1.5 [1.3–1.7]	<0.0001	1.4 [1.1–1.7]	0.0015
**Diabetes mellitus**	1.6 [1.4–1.8]	<0.0001	1.9 [1.6–2.4]	<0.0001
**Renal disease**	1.0 [0.7–1.3]	0.8166	1.0 [0.7–1.6]	0.8486
**Cancer**	1.1 [0.8–1.4]	0.5013	1.5 [1.1–2.2]	0.0210

*Abbreviations: SA = sleep apnea; CCI = Charlson Comorbidity Index; HR = hazard ratio; CI = confidence interval.

Sensitivity analyses showed consistent results (Table D in S1 File).

## Discussion

This large population-based cohort study revealed that patients with SA had significantly higher incidence of ED than subjects without ED. The analyses using SA patients identified with merely the diagnostic codes (i.e., suspected SA cohort in study arm A) and the analyses using SA patients identified with the presence of diagnosis after PSG (i.e., probable SA cohort in study arm B) showed consistent results. Multivariable Cox regression analyses showed that SA remained an independent risk factor for developing ED after adjusting for age and comorbidities.

To the best of our knowledge, this is the first long-term nationwide population-based cohort study to investigate ED incidence in SA patients. Many studies to date have demonstrated the association between SA and ED since 1990, when Hirshkowitz *et al*. reported 43.8% of ED patients had apnea index ≥5 [6,14–20]. A study using questionnaires in a health-screening program showed that both SA and ED were prevalent and might be related to each other in adult men [17]. Other studies using questionnaires also found that the ED risk was significantly higher in patients with severe obstructive SA and the correlation between the severity of SA and the severity of ED was strong [14,16]. A case-control study further showed that obstructive SA patients had significantly more ED and sexual dissatisfaction as compared with age-matched control [25]. However, it had remained uncertain whether the association between SA and ED was maintained in the presence of other risk factors for ED until a prospective cross-sectional analysis by Budweiser *et al*. confirmed that SA was an independent risk factor for ED after adjusting for many known risk factors, such as age and hypertension [19]. A population-based cross-sectional study in Sao Paolo, Brazil also showed obstructive SA was significantly associated with a higher risk of ED [26]. Although many studies have been done in discussing the relationship between SA and ED, these studies are cross-sectional studies and are relatively small in size. Our population-based study not only showed results consistent with the previous studies, but also demonstrated a temporal relationship using a cohort study design. The sensitivity analyses using “incubation time” design further strengthened the evidence showing the association between SA and ED. In line with previous studies, we found old age, diabetes mellitus, hypertension, and liver diseases were also independent risk factors for ED from the multivariable Cox regression analysis [26–28]. Interestingly, the effect of SA was less significant in the elder patients and patients without comorbidities than in younger patients and patients with comorbidities, respectively. The findings might be explained by possible less awareness of sexual dysfunction in the elder population and patients with comorbidities in Taiwan. However, as a more reasonable explanation in our opinion, the ED risk had already been increased by aging or comorbidities, so the effect of SA was somewhat obscured. Further prospective studies are needed to clarify this point.

Intermittent hypoxia, a hallmark of SA, plays an important role in the health effect of SA, and ED is no exception. A previous study showed that the degree of hypoxemia correlated with the score on a questionnaire-based scale evaluating erectile function [29]. The role of hypoxia was also well demonstrated by an animal study showing that chronic intermittent hypoxia during sleep was associated with emergence of ED and decreased libido in mice [30]. Indeed, treating with continuous positive airway pressure (CPAP), the gold standard in treating SA, may also improve the sexual performance in patients having both SA and ED [18,25,31–34]. Other treatment strategies for SA, such as oral appliances (mandibular advancement devices) and surgical intervention (uvulopalatopharyngoplasty), might also improve ED [25,35]. Some studies suggested that sildenafil was superior to CPAP in treating ED in SA patients [31,34,36,37]. However, due to the detrimental effect of sildenafil on respiratory and desaturation events during sleep [38], this drug should be used carefully. Combination of CPAP and oral sildenafil might be a more effective and safer treatment modality for ED in SA patients [36,37].

The pathogenic mechanisms contributing to the development of ED in SA patients, including abnormalities in neural, hormonal and vascular regulation, have been discussed [20]. The development of peripheral nerve dysfunction, as shown by altered bulbocavernosus reflex, was associated with ED in SA patients [39]. Although low testosterone level was associated with ED, studies showed that testosterone level was not significantly changed in patients receiving treatment for SA or in mice treated with intermittent hypoxia [25,26,30,40]. In the recent decade, much attention has been paid to the role of endothelial dysfunction, which is also one of the main mechanisms for cardio-metabolic consequences of SA [6]. A study of serum inflammatory markers and cytokines showed elevated serum concentration of high-sensitivity C-reactive protein, tumor necrosis factor-α (TNF-α), interleukin-6 (IL-6) and interleukin-8 (IL-8) in SA patients with ED, as compared with those without ED; this study supported the possible involvement of endothelial dysfunction in the development of ED in SA patients [41]. The study using murine model also found decreased expression of nitric oxide synthase (NOS) in erectile tissue after chronic intermittent hypoxia [30]. In addition, a rat study showed that long-term intermittent hypoxia increased nicotinamide adenine dinucleotide phosphate (NADPH) oxidase activation, which decreased the expression and activity of constitutive NOS, including endothelial NOS and neuronal NOS, and thereby contributed to the development of ED [42]. Taken together, endothelial dysfunction related to intermittent hypoxia might be the most important mechanism underlying ED related to SA. Since CPAP has been demonstrated to be able to reverse endothelial dysfunction in SA patients [43], it is not surprising that CPAP treatment improves sexual function in patients having both SA and ED [18,25,31–34].

Our study has two major strengths. First, our study is the largest study to date discussing about the association between SA and ED. As compared with lab-based or hospital-based setting, the nationwide, population-based setting minimizes selection bias and provides a broad view of the real world. Second, this is a cohort study with a long follow-up period. As compared with the previous studies using cross-sectional design, our study provides better evidence about the association between SA and ED by demonstrating a temporal trajectory.

Nevertheless, this study had several limitations. First, the diagnosis based on diagnostic codes might be less accurate than those made according to standardized criteria in clinical trials. Therefore, in addition to the analyses using SA patients identified with merely the diagnostic codes (i.e., suspected SA cohort), we performed another set of analyses using SA patients having SA diagnosis after PSG (i.e., probable SA cohort) and found consistent results. Besides, the method using diagnostic codes to identify ED in the NHI database has been used in many previous studies [22,23]. Second, it is not possible to adequately distinguish OSA and CSA based on the diagnostic codes in our database. It is therefore not possible to assess whether OSA and CSA affect the risk of ED in the same way. However, because the vast majority of SA is OSA, it is OSA that might substantially increase the risk of ED [1,2]. Third, some well-known potentially important clinical risk factors for ED, such as smoking history, obesity, and alcoholism, were not available in the NHI database, so interpretation of our results must be careful to account for possible impact of these risk factors. Nevertheless, SA remained an independent risk factor even in the multivariable Cox regression analyses adjusting for various comorbidities, which minimized the effect of these confounders. Fourth, because SA patients seemed having higher income level, this could potentially introduced a bias toward more awareness to sexual dysfunction in the higher socio-economic group and thus leaded to higher incidence of ED in the SA cohorts. We therefore incorporated the residency and income level into the multivariable models to control these possible confounders. Finally, information about the severity of SA and major treatment modalities, such as CPAP and oral appliances, were not available in the NHI database. However, the inclusion of milder SA patients and SA patients receiving treatment might abate the extent of increased ED risk from SA, resulting in underestimation of the hazard ratios in our study.

In conclusion, the present large nationwide population-based cohort study confirms SA as an independent risk factor for ED. Clinicians should consider ED while seeing patients with SA, and men with ED might also benefit from sleep evaluation.

## Supporting Information

S1 File Table A. Diagnostic codes used for identifying sleep apnea. **Table B.** Multivariable Cox regression analysis of the factors contributing to erectile dysfunction (reduced models of study arm A). **Table C.** Multivariable Cox regression analysis of the factors contributing to erectile dysfunction (reduced models of study arm B). **Table D.** Sensitivity analyses.
(PDF)
