# Supplementary material for: Erectile Dysfunction in Patients with Sleep Apnea – A Nationwide Population-Based Study
Source: PLoS One. 2015 Jul 15;10(7):e0132510. doi: 10.1371/journal.pone.0132510 (PMC4503619; doi:10.1371/journal.pone.0132510)
Supplement: S1 File — Table B. Multivariable Cox regression analysis of the factors contributing to erectile dysfunction (reduced models of study arm A). Table C. Multivariable Cox regression analysis of the factors contributing to erectile dysfunction (reduced models of study arm B). Table D. Sensitivity analyses. (PDF) [file pone.0132510.s001.pdf]

Erectile Dysfunction in Patients with Sleep Apnea – A Nationwide Population-Based Study

Supporting Information

S1 File

Authors:

Chia-Min Chen[1¶], Ming-Ju Tsai[1,2,3¶], Po-Ju Wei[1¶], Yu-Chung Su[4,5], Chih-Jen Yang[1,2,4,5], Meng-Ni Wu[3,6], Chung-Yao Hsu[3,5,6], Shang-Jyh Hwang[7,8], Inn-Wen Chong[1,9], Ming-Shyan Huang[1,2,5,10 *]

[1] Division of Pulmonary and Critical Care Medicine, Department of Internal Medicine, Kaohsiung Medical University Hospital, Kaohsiung Medical University, Kaohsiung, Taiwan

[2] Graduate Institute of Medicine, College of Medicine, Kaohsiung Medical University, Kaohsiung, Taiwan

[3] Sleep Disorders Center, Kaohsiung Medical University Hospital, Kaohsiung Medical University, Kaohsiung, Taiwan

[4] Department of Internal Medicine, Kaohsiung Municipal Ta-Tung Hospital, Kaohsiung Medical University, Kaohsiung, Taiwan

[5] School of Medicine, College of Medicine, Kaohsiung Medical University, Kaohsiung, Taiwan

[6] Department of Neurology, Kaohsiung Medical University Hospital, Kaohsiung Medical University, Kaohsiung, Taiwan

[7] Division of Nephrology, Department of Internal Medicine, Kaohsiung Medical University Hospital, Kaohsiung Medical University, Kaohsiung, Taiwan

[8] Department of Renal Care, College of Medicine, Kaohsiung Medical University, Kaohsiung, Taiwan

[9] Department of Respiratory Care, College of Medicine, Kaohsiung Medical University, Kaohsiung, Taiwan

[10] Division of Geriatrics and Gerontology, Department of Internal Medicine, Kaohsiung Medical University Hospital, Kaohsiung Medical University, Kaohsiung, Taiwan

[¶] These authors contributed equally to this work.

[*] Corresponding author
E-mail: shyan310@gmail.com (MSH)

Table A. Diagnostic codes used for identifying sleep apnea.

ICD-9-CM code	Diagnosis
327.20	Organic sleep apnea, unspecified
327.21	Primary central sleep apnea
327.23	Obstructive sleep apnea (adult)(pediatric)
327.27	Central sleep apnea in conditions classified elsewhere
327.29	Other organic sleep apnea
780.51	Insomnia with sleep apnea, unspecified
780.53	Hypersomnia with sleep apnea, unspecified
780.57	Unspecified sleep apnea

Table B. Multivariable Cox regression analysis of the factors contributing to erectile dysfunction (reduced models of study arm A).

	Reduced model 1	Reduced model 2	Reduced model 3	Reduced model 4	Reduced model 5	Reduced model 6	Reduced model 7	Reduced model 8
**SA patients vs. Control subjects**	1.8 [1.5-2.2]***	1.9 [1.6-2.3]***	1.9 [1.6-2.4]***	1.9 [1.6-2.3]***	1.8 [1.5-2.2]***	1.6 [1.3-2.0]***	1.7 [1.4-2.1]***	1.8 [1.5-2.2]***
**Age > 50 vs. Age ≤ 50**	3.0 [2.7-3.3]***	2.8 [2.5-3.1]***	2.8 [2.6-3.1]***		2.5 [2.2-2.7]***	2.6 [2.3-2.9]***	2.4 [2.2-2.7]***	2.4 [2.2-2.7]***
**Residency (Northern Taiwan vs. Other areas)**	1.4 [1.3-1.5]***					1.4 [1.3-1.6]***		
**Higher income (> NT$24000) vs. lower income (≤ NT$24000)**	1.4 [1.3-1.6]***					1.4 [1.3-1.6]***		
**Heart disease (with vs. without)**	0.8 [0.6-1.1]	0.8 [0.6-1.1]	0.8 [0.6-1.1]	1.3 [0.9-1.8]				
**Major neurological disorder (with vs. without)**	1.2 [1.0-1.4]	1.1 [0.9-1.3]						
**Any comorbidity (CCI score ≥1 vs. = 0)**					1.5 [1.3-1.6]***			
**Diabetes mellitus (with vs. without)**						1.5 [1.3-1.7]***	1.4 [1.2-1.7]***	1.5 [1.3-1.8]***
**Hypertension (with vs. without)**						1.3 [1.1-1.4]***	1.2 [1.1-1.4]**	1.3 [1.1-1.4]***
**Liver diseases (with vs. without)**						1.5 [1.3-1.7]***	1.5 [1.3-1.7]***	

Abbreviations: SA = sleep apnea; CCI = Charlson Comorbidity Index.

Data presented are hazard ratio [95% confidence interval].

**p*<0.05; ***p*<0.01; ****p*<0.0001

Table C. Multivariable Cox regression analysis of the factors contributing to erectile dysfunction (reduced models of study arm B).

	Reduced model 1	Reduced model 2	Reduced model 3	Reduced model 4	Reduced model 5	Reduced model 6	Reduced model 7	Reduced model 8
**SA patients vs. Control subjects**	2.2 [1.7-2.9]***	2.4 [1.8-3.2]***	2.4 [1.8-3.2]***	2.4 [1.8-3.2]***	2.1 [1.6-2.8]***	1.9 [1.5-2.6]***	2.1 [1.6-2.8]***	2.2 [1.6-2.9]***
**Age > 50 vs. Age ≤ 50**	2.7 [2.3-3.1]***	2.5 [2.2-3.0]***	2.6 [2.2-3.0]***		2.2 [1.9-2.6]***	2.2 [1.9-2.6]***	2.1 [1.8-2.5]***	2.1 [1.8-2.5]***
**Residency (Northern Taiwan vs. Other areas)**	1.4 [1.2-1.6]***					1.4 [1.2-1.6]***		
**Higher income (> NT$24000) vs. lower income (≤ NT$24000)**	1.3 [1.1-1.5]**					1.3 [1.1-1.5]**		
**Heart disease (with vs. without)**	1.1 [0.7-1.7]	1.1 [0.7-1.6]	1.1 [0.7-1.8]	1.7 [1.1-2.6]*				
**Major neurological disorder (with vs. without)**	1.5 [1.2-2.0]**	1.4 [1.1-1.9]*						
**Any comorbidity (CCI score ≥1 vs. = 0)**					1.9 [1.6-2.2]***			
**Diabetes mellitus (with vs. without)**						1.7 [1.4-2.1]***	1.7 [1.4-2.1]***	1.8 [1.4-2.5]***
**Hypertension (with vs. without)**						1.5 [1.3-1.8]***	1.5 [1.3-1.8]***	1.5 [1.3-1.8]***
**Liver diseases (with vs. without)**						1.4 [1.1-1.7]**	1.4 [1.2-1.7]**	

Abbreviations: SA = sleep apnea; CCI = Charlson Comorbidity Index.

Data presented are hazard ratio [95% confidence interval].

*p<0.05; **p<0.01; ***p<0.0001

Table D. Sensitivity analyses.

Sensitivity analyses	Control cohort				SA cohort				Crude IRR [95% CI]	Adjusted IRR [95% CI]	aHR [95% CI]
	N	ED	PY	IR	N	ED	PY	IR			
**Study arm A (suspected SA vs. control A)**											
**Analysis_A1**	48100	405	186732.8	2.2	4810	82	18605.7	4.4	2.0 [1.9-2.2]***	1.8 [1.6-1.9]***	1.8 [1.4-2.3]***
**Analysis_A2**	39790	285	142629.5	2.0	3979	64	14193.4	4.5	2.3 [2.1-2.4]***	2.0 [1.8-2.2]***	2.0 [1.5-2.6]***
**Analysis_A3**	32490	197	106214.9	1.9	3249	44	10573.8	4.2	2.2 [2.0-2.5]***	1.8 [1.7-2.0]***	1.8 [1.3-2.6]**
**Analysis_A4**	25760	132	76616.0	1.7	2576	34	7633.5	4.5	2.6 [2.3-2.9]***	2.4 [2.1-2.6]***	2.4 [1.6-3.5]***
**Analysis_A5**	19650	89	53850.9	1.7	1965	22	5368.0	4.1	2.5 [2.2-2.8]***	2.3 [2.1-2.6]***	2.3 [1.4-3.7]**
**Study arm B (probable SA vs. control B)**											
**Analysis_B1**	19330	148	72992.2	2.0	1933	40	7247.9	5.5	2.7 [2.4-3.0]***	2.2 [2.0-2.5]***	2.2 [1.6-3.2]***
**Analysis_B2**	16090	97	55160.4	1.8	1609	32	5465.2	5.9	3.3 [3.0-3.7]***	2.9 [2.5-3.2]***	2.9 [1.9-4.4]***
**Analysis_B3**	13040	75	40456.2	1.9	1304	22	4007.6	5.5	3.0 [2.6-3.4]***	2.5 [2.1-2.8]***	2.4 [1.5-4.0]**
**Analysis_B4**	10010	54	28595.3	1.9	1001	15	2844.6	5.3	2.8 [2.4-3.3]***	2.7 [2.3-3.2]***	2.7 [1.5-5.0]**
**Analysis_B5**	7370	32	19811.2	1.6	737	10	1975.3	5.1	3.1 [2.6-3.7]***	2.5 [2.1-3.0]***	2.5 [1.2-5.2]*

Sensitivity analyses:

Analysis_A1-A5: After excluding the subjects having events in the initial 1-5 years of the follow-up period, the sensitivity analyses were performed on the remaining suspected SA patients and ten randomly-selected corresponding control A subjects with the new follow-up periods starting from 1-5 years after the index dates.

Analysis_B1-B5: After excluding the subjects having events in the initial 1-5 years of the follow-up period, the sensitivity analyses were performed on the remaining probable SA patients and ten randomly-selected corresponding control B subjects with the new follow-up periods starting from 1-5 years after the index dates.

The adjusted IRRs were calculated by multivariable analyses adjusting for age, residency, income and the presence of various comorbidities.

The adjusted hazard ratios (aHRs) of sleep apnea were calculated by multivariable analyses adjusting for age, residency, income and the presence of various comorbidities.

*$p<0.05$; **$p<0.01$; ***$p<0.0001$

Abbreviation: SA = sleep apnea; CCI = Charlson Comorbidity Index;
N = number of patients; ED = number of patients with erectile dysfunction; PY = total patient-years;
IR = incident rate, as expressed as ED incidence per 1000 patient-years; IRR = incidence rate ratio; CI = confidence interval.
